# Effectiveness and cost-effectiveness of a patient-initiated botulinum toxin treatment model for blepharospasm and hemifacial spasm compared to standard care: study protocol for a randomised controlled trial

**DOI:** 10.1186/s13063-016-1263-y

**Published:** 2016-03-09

**Authors:** Sadie Wickwar, Hayley McBain, Stanton P. Newman, Shashivadan P. Hirani, Catherine Hurt, Nicola Dunlop, Chris Flood, Daniel G. Ezra

**Affiliations:** Centre for Health Services Research, School of Health Sciences, City University London, London, UK; Moorfields Eye Hospital, London, UK; Community Health Newham, East London Foundation Trust, London, UK

**Keywords:** Blepharospasm, Hemifacial spasm, Botulinum toxin, Patient-led care, Quality of life, Illness perceptions, Randomised controlled trial, Protocol, Acceptability

## Abstract

**Background:**

Blepharospasm and hemifacial spasm are debilitating conditions that significantly impact on patient quality of life. Cyclical treatment with botulinum toxin injections offers temporary relief, but the duration of treatment efficacy is variable. The standard model of patient care defines routine fixed-time based scheduled treatment cycles which may lead to unnecessarily frequent treatment for some patients and experience of distressing symptoms in others, if symptoms return before the scheduled follow-up period.

**Methods/Design:**

A randomised controlled trial will compare a patient-initiated model of care, where patients determine botulinum toxin treatment timing, to the standard model of care in which care is scheduled by the clinical team. A sample of 266 patients with blepharospasm or hemifacial spasm will be recruited from Moorfields Eye Hospital (MEH), London. The trial will be accompanied by a mixed-methods evaluation of acceptability of the new service. Patients who meet eligibility criteria will be assessed at baseline and those in the intervention group will be provided with instructions on how to book their own treatment appointments. Patients in both groups will be followed up 3 and 9 months into the trial and all patients will be returned to usual care after 9 months to meet safety protocols. Primary outcome measures include disease severity (questionnaire), functional disability (questionnaire) and patient satisfaction with care (questionnaire). Secondary outcomes include disease-specific quality of life (questionnaire), mood (questionnaire), illness and treatment perceptions (questionnaire and semi-structured interviews), economic impact (questionnaire) and acceptability (questionnaire and semi-structured interviews).

**Discussion:**

This trial will assess the effectiveness and cost-effectiveness of a patient-led care model for botulinum toxin therapy. If the new model is shown to be effective in reducing distress and disability in these populations and is found to be acceptable to patients, whilst being cost-effective, this will have significant implications for service organisation across the NHS.

**Trial registration:**

UK Clinical Research Network (UKCRN) Portfolio 18660. Clinicaltrials.gov ID NCT102577224 (registered 29 October 2015)

## Background

Blepharospasm is a dystonia described by sustained, forced, involuntary closure of both eyelids, caused by muscle contraction. Hemifacial spasm occurs on one side of the face and can result in complete closure of one eye, and spasms across the cheek, face and neck. Hemifacial spasm, as its name implies, is unilateral, whereas blepharospasm always affects both eyes. Blepharospasm and hemifacial spasm are debilitating conditions, which carry the risk of functional blindness and can lead to appearance concerns, social embarrassment and isolation, depression and poor quality of life [1–3].

Botulinum toxin is the treatment used in standard care to alleviate spasms, but by their nature result in a cyclical pattern of relief and aggravation as the toxin wears off [4] and hence patients return for repeated injections. A recent systematic review indicated that the patient- reported benefits of botulinum toxin in blepharospasm ranged from no improvement to 96 % of patients reporting a significant relief in symptoms [5]. Evidence for the duration of benefit provided by repeated treatment is also markedly inconsistent [5]. Although the definition of benefit did differ between studies in this review, much of the inconsistency between studies remains unresolved. Despite this, a dosing interval of 3- to 4-monthly injections is typical for many different dystonias across the UK. However, the variation in response to this treatment may mean that some people are left experiencing debilitating symptoms until their next scheduled appointment and some are being seen more often than their symptoms would deem necessary. It would potentially be more useful, therefore, to explore alternative models of care based more closely on symptoms.

Patient-centred care is at the forefront of the NHS [6, 7] reflecting the shift away from the paternalistic model of healthcare. Patients are now encouraged to take a more active role in knowing and managing their health, and this is especially important in conditions such as dystonia where the reality of living with the condition is demanding. As a result patient-led healthcare services are becoming increasingly more common [8] and have the potential to address the inconsistencies found in the current botulinum toxin treatment regimen.

Studies of patient-initiated services, where the patient rather than healthcare professional initiates treatment and care, have shown promise. These services provide patients with information on when and how to access services, rather than having regular time-based scheduled appointments. A systematic review conducted by Whear et al. [8] synthesised the evidence for this model of care across three conditions: irritable bowel disease, breast cancer and rheumatoid arthritis. Overall, there were few differences in psychological or health-related quality of life outcomes between those initiating their own outpatient follow-up appointments compared to standard care, despite in many cases patients having less contact with healthcare professionals. Patient and clinician satisfaction were significantly greater in the patient-initiated services compared to regular appointment scheduling. Another more recent trial has also found significant reductions in healthcare utilisation without compromising clinical or psychosocial well-being [9].

Adopting a patient-led model of care has the potential to reduce morbidity and disability in patients with a short-term response to botulinum toxin. Conversely, this may also reduce unnecessary hospital visits and treatment for patients with a longer-term response. There is currently one patient-initiated, nurse-led botulinum toxin clinic running in the UK for patients with blepharospasm or hemifacial spasm [10], but is yet to be evaluated in comparison to usual care. Due to the variable nature of the duration of response to treatment, blepharospasm and hemifacial spasm are appropriate conditions in which to evaluate a service of this nature. This study, therefore, provides a unique opportunity to empower patients with dystonia to take control of their treatment scheduling and optimise the effects of botulinum toxin, by allowing them to seek treatment when they feel it is necessary rather than it being dictated by the clinical team.

The primary aims of this randomised controlled trial (RCT) are to: (1) investigate the effectiveness of a patient-led model for botulinum toxin treatment in maintaining a more stable pattern of disease severity and disability in patients with hemifacial spasm and blepharospasm in comparison to standard care, and (2) assess patient satisfaction with the new treatment model compared to standard care. The secondary aims are the assessment the impact of the service on psychosocial outcomes, including quality of life, illness perceptions, mood, acceptability and cost-effectiveness.

## Methods

### Study design

This study will be mixed methods adopting a concurrent embedded strategy [11], whereby a qualitative study will be embedded within a larger single-masked RCT, with the RCT as the dominant component. This design will provide information on effectiveness and also an in-depth assessment of the patient experience. The RCT will be a parallel-group, explanatory, superiority RCT designed to assess the effectiveness of a patient-initiated botulinum toxin service compared to usual care. Semi-structured interviews will be conducted with a sample of intervention participants in order to assess acceptability of the service.

### Randomisation

Randomisation (1:1) will be undertaken by a central randomisation service by the data management team at Moorfields Eye Hospital (MEH) in order to ensure the research fellow collecting and analysing the data is blinded to group allocation. Participants will be randomised to receive either treatment as usual or patient-initiated follow-up appointments. Randomly permutated blocks of varying sizes will be employed to ensure balance between treatment groups. Due to the nature of the nurse-led botulinum toxin clinic it will not be possible to blind the treating healthcare professionals from group allocation. The trial co-ordinator will staff the telephone line that participants in the intervention group will use to book their appointments and, as such, the trial co-ordinator will not be blinded to group allocation.

### Blinding

Due to the nature of the study, participants cannot be blinded to group allocation. The treating healthcare professionals will also not be masked. The research fellow responsible for entering data into the database and the statistician conducting data analysis will be blinded to participant allocation. All other members of the research team will be blinded to allocation throughout the study, prior to un-blinding at the end of analysis.

### Setting

The outpatient nurse-led botulinum toxin clinic at MEH.

### Inclusion criteria

Patients aged 18 years or over who have received a consultant-led diagnosis of hemifacial spasm or blepharospasm will be invited to take part in the trial. Eligible patients need to be on a stable dose of botulinum toxin treatment, defined as receiving toxin treatment over two previous cycles and free from side effects. Potential side effects of botulinum toxin treatment include ptosis, double or blurred vision, and foreign body sensations. Patients must possess the capacity to give informed consent to participate in the study, as judged by the specialist research nurse leading the clinic.

### Exclusion criteria

Patients will be excluded if they have significant co-morbidities (i.e. their predominant treatment is for another illness) and/or they are unable to communicate fluently in written and/or spoken English, to complete study measures.

### Intervention

Participants randomised to the intervention group will initiate their own treatment during the trial period (9 months). They will be given information about when and how to initiate an appointment in the nurse-led botulinum toxin clinic, in a leaflet sent to them by a trial co-ordinator after randomisation. Participants will be asked to contact the service when they feel their symptoms are returning at a sufficient level for them to seek medical help. Contact details for the service will also be provided along with information on how quickly an appointment will be made, with whom, and the procedure in the case of an emergency. Receipt of the leaflet will be followed by a telephone call from one of the research team to answer any questions participants may have. When participants in the intervention group contact the service to book an appointment they will be triaged by the trial co-ordinator. All patients with an activity score of 1 or above on the Jankovic Rating Scale (JRS; [12]) will be booked in to the next available slot within the twice-weekly nurse-led outpatient clinics, estimated within a 2-week period from the initial call. There will be no upper limit for the number of times participants in the intervention group can initiate an appointment. However, participants will be advised to wait 2 weeks after botulinum toxin treatment as this is the time it takes to reach effectiveness. It is, therefore, estimated that participants would not return to the clinic within 3 to 4 weeks of their previous appointment. All participants will be given an appointment at 9 months to meet safety criteria for any patients who do not seek an appointment during their participation in the trial.

#### Control group

Participants in the control group will receive usual care. This consists of scheduled appointments in the nurse-led outpatient botulinum toxin clinic, usually every 3 months.

### Outcomes

Participants will be in the trial for 9 months. An initial baseline assessment will be taken by a research fellow prior to randomisation; this will include the full range of measures. Self-report measures will be taken again at 3- and 9-month follow-ups.

At baseline, demographic information will be collected using self-report measures including: date of birth, gender, marital status, ethnicity, postcode, number of years of schooling, highest completed level of education and further education. Data will also be collected from the patients’ medical notes on diagnosis, year of diagnosis, duration of botulinum toxin treatment, number of previous cycles, frequency of previous cycles, last dose, and co-morbidities. The total number of botulinum toxin injections and dosage received by the patient across the trial period will be collected.

#### Primary outcome measures

##### Disease severity and symptoms

Disease severity and frequency of symptoms will be assessed using the JRS [12], a clinician-reported measure that consists of two subscales measuring symptom severity and frequency independently. A 2-point improvement in the JRS sum score is considered a clinically relevant improvement [13]. However, these criteria are only relevant for patients whose baseline scores are >2 on the JRS [13]. For patients with hemifacial spasm an additional rating scale for severity and frequency of cheek involvement will be included in the JRS, as suggested by Wabbels and Roggenkämper [14].

##### Functional disability

The Blepharospasm Disability Index (BSDI)© [15, 16] is a patient self-report measure that asks about six daily activities: reading, driving a vehicle, watching TV, shopping, walking and doing everyday activities. The measure is also recommended for use in hemifacial spasm [14]. Each activity is rated on a scale from 0 = no impairment to 4 = no longer possible due to my illness, and a ‘not applicable’ option is also available. The scoring system is a mean item score, calculated by dividing the sum score by the number of applicable items. Sum scores, therefore, range from 0 to 4 with higher scores indicating greater disability. A 0.7-point improvement in the BSDI© mean item score is considered a clinically relevant improvement [13]. However, this criteria is only relevant for patients whose baseline scores are >0.7 [13]. The measure possesses good convergent validity with the JRS, good internal consistency (Cronbach’s alpha = 0.88) and adequate test-retest reliability [13].

##### Patient satisfaction

Patient satisfaction will be measured using the eight-item Client Satisfaction Questionnaire (CSQ; [17]). Responses to each of these eight items range on a Likert scale from 1 to 4, each item has different weighted responses and patients will be instructed to answer in response to the care they receive in the nurse-led botulinum toxin clinic. Items include ‘How would you rate the overall quality of the service your received?’. Scale scores are a sum of the eight items and range from 8 to 32, with higher scores indicating greater satisfaction. The scale has good internal reliability, with a Cronbach’s alpha of 0.93 and good construct validity [18].

#### Secondary outcome measures

##### Quality of life

Quality of life will be measured using the Craniocervical Dystonia Questionnaire (CDQ-24; [19]), which was developed and validated in patients with blepharospasm and has also been used with success in patients with hemifacial spasm [20]. This 24-item measure assesses quality of life across five domains: stigma, emotional well-being, pain, activities of daily living and social/family life. Each item consists of five statements representing increasing severity of impairment and is scored from 0 to 4. In order to obtain comparable scores for the individual subscales, raw sub-scores (= sum of the individual item score) are linearly transformed to a 0–100 scale, where a score of 0 indicates the best and a score of 100 the worst possible quality of life. The measure has been found to possess good internal consistency with Cronbach’s alphas ranging from 0.77 to 0.89, good construct validity when compared who the Short Form 36 (SF-36), good discriminant validity and test-retest reliability [20].

##### Mood

The Hospital Anxiety and Depression Scale (HADS; [21]) will be used to assess mood. The HADS is a 14-item self-screening questionnaire for depression and anxiety in patients with physical health problems and the two 7-item subscales measure how the respondent has been feeling in the past week. The scale scores range from 0 to 21, with higher scores indicating greater levels of anxious or depressed mood. A score of 0–7 on either subscale is regarded as being in the ‘normal’ range, a score of 8–10 is suggestive of the presence of moderate levels of anxiety or depression, and a score of 11 or above indicates ‘caseness’, a high likelihood that a person would be diagnosed with clinical anxiety or clinical depression. A systematic review of the HADS has confirmed the factor structure and found the cut-off points to be valid against clinical interviews [22]. This tool has demonstrated high internal consistency (*r* = 0.76 to 0.41 for anxiety scale items and *r* = 0.60 to 0.30 for depression scale items) and good reliability [23].

##### Illness beliefs

Illness perceptions are cognitive representations or beliefs that a patient has about their illness. These concepts will be measured using the revised Illness Perceptions Questionnaire Revised (IPQ-R; [24]) that assesses each of the components of illness representations (Table 1).

**Table 1:** Definitions of the Illness Perceptions Questionnaire Revised (IPQ-R) subscales

Subscale	A belief…..
Cause	…about the cause of my illness
Identity	…about the number of symptoms attributable to their condition
Time (acute/chronic)	…about the duration of the condition
Consequences	…that their condition will have serious consequences
Personal control	…in one’s ability to personally influence the outcome of their condition
Treatment control	…that medical treatments will be effective in controlling their condition
Illness coherence	…that condition ‘makes sense’
Timeline cyclical	…that the condition will come and go in cycles
Emotional representations	…that the condition is emotionally distressing

Total scores on the illness identity subscale range from 0 to 14 and higher scores represent strong beliefs about the number of symptoms attributed to their condition. In order to increase the face validity of this subscale four blepharospasm and hemifacial spasm symptoms were added to the identity scale: (1) frequent blinking, (2) irritation of the eye, (3) uncontrollable eye closure and, and (4) muscle twitching around the face and/or eye; bringing the maximum score up to 18. Total scores range from 6 to 30 for the consequences, timeline acute/chronic, emotional representation and personal control subscales and 5–25 for treatment control and illness coherence and 5–20 for timeline cyclical. High scores represent strongly held beliefs about their condition. The subscales have been found to possess good internal consistency [25]. Cause is assessed in 18 items which are analysed in three groupings, causes relating to psychological attributions, risk factors and immunity. Each of these subscales possesses good internal consistency ranging from 0.67 for immunity to 0.86 for the psychological attribution subscale [25]. Participants are also asked to rank the three most important factors that they believe caused their illness.

##### Treatment beliefs

Beliefs about botulinum toxin will be measured using the Treatment Representations Inventory (TRI; [21]); a 27-item measure, consisting of four scales measure on a 5-point Likert agreement scale. Scales include treatment-value, treatment-concerns, decision-satisfaction and cure. Scales demonstrate good internal consistency (Cronbach’s alpha: 0.78, 0.77, 0.80 and 0.75 respectively).

##### Confidence

Confidence in the service will be assessed using a 10-cm visual analogue scale (VAS), as in other evaluations of patient-initiated services [24, 26, 27]. Participants will be asked ‘How confident are you that if you required treatment this system of care would be able to support you?’, ranging from ‘not at all confident’ to ‘completely confident’. Individual side effects will also be recorded at each clinic visit by the treating healthcare professional along with a subjective assessment of the duration of beneficial effect of their last treatment, in weeks.

##### Acceptability

Acceptability of the new patient-initiated service and standard care will be measured using the seven-item Acceptability Questionnaire [28]. Responses are on a Likert scale from 1 to 5 and response labels vary depending on the item, for example, ‘How fair do you feel this system has been?’ has the response options ‘not at all fair’ (1) to ‘very fair’ (5). The questionnaire will be validated using established methods once all data has been collected.

##### Health-care usage

Use of health and social care services will be estimated using a brief version of the Client Service Receipt Inventory (CSRI; [29]) completed by a combination of self-report and electronic patient records (EPR). The CSRI has been validated and widely employed in previous studies, particularly in the mental health setting [29] and has been found to be a suitable tool for use with patients experiencing psychological distress [30].

##### Cost-effectiveness

The impact of the service on direct and indirect costs will be estimated at the end of the trial period using this brief CSRI [29]. This data will be collected by the research nurse at the 9- month follow-up visit, who will ask participants to report which services they have accessed in the past 9 months. This information will be cross-referenced with each patient’s hospital notes to minimise recall bias. The accurate collection of resource-use data is commonly a challenge in economic evaluation and the method of comparing patient self-report to healthcare records has been used in recent research [31]. Unit costs of healthcare resources will be derived from the NHS trust where possible. For sensitivity analysis, and/or where there is an absence of costs available locally, national unit costs that are judged representative of local costs will be obtained from national sources, such as NHS Commissioning or NHS England. Costs associated with the different resources and services will be reported as part of this economic evaluation. To calculate the costs, we will look at: (1) resource use in primary care, including an examination of total botulinum toxin used, visits to the GP, and other services, (2) resource use in secondary care including attendance at accident and emergency clinics (A&E), (3) resource use from specialty services, (4) intervention costs and costs of treatment as usual for comparison, (5) resources associated with any admissions will be recorded and totalled with all the previously identified resources, to calculate a total cost to the health service overall, (6) resource use in social care including social worker and community psychiatric nurse, and (7) out-of-pocket costs to the individual such as visual aids and travel to appointments. Out-of-pocket costs, including the cost of visual aids, to the individual will also be recorded at baseline. A fuller description of the methods used to conduct the cost-effectiveness analysis is described in the statistical methods section.

##### Frequency of outpatient visits

The length of time between visits to the nurse-led clinic will be recorded (in days) for participants in both arms of the trial. In the patient-initiated treatment group the reasons for initiating a consultation will be recorded at the time of initiating the appointment.

##### Adverse effects

The frequency of adverse events including ptosis, double or blurred vision, tearing, hematoma and foreign body sensation will also be recorded at each clinic visit as either present or absent. Patients will also be followed up 2 weeks after each visit in which they receive botulinum toxin treatment to assess any adverse events.

### Procedures

Invitation letters will be sent to eligible patients 2 weeks prior to their outpatient appointment in the nurse-led botulinum toxin clinic by the trial co-ordinator. On the day of the appointment the patient will be approached by the research fellow to participate in the study. Those who agree will be consented into the trial by the research fellow. Qualitative data will be collected on reasons for refusal. Baseline assessment questionnaires will be given to participants consenting to take part in the study with a freepost envelope for return, all subsequent follow-up self-report questionnaires will be sent to the participants’ home also with a freepost envelope for return. Participants who do not return questionnaires within 2 weeks will be contacted by telephone. If participants do not respond to one follow-up telephone call and one letter, and there is no evidence that the patient has moved, it will be considered that they no longer wish to take part in the trial and they will not be sent further questionnaires. They will continue in the trial on the basis that clinical and cost-effectiveness data can still be collected and an intention-to-treat analysis (ITT) will be performed. Participants in the intervention trial arm who no longer wish to take part in the trial but are happy to continue to complete questionnaires will also be included in the ITT analysis.

Disease severity and function will be assessed at baseline and then at each clinic visit by the treating healthcare professionals and again 2 weeks after treatment via telephone by the research fellow.

Participants in the intervention group who have agreed to be contacted about an interview to assess acceptability of the new service will be followed up with a telephone call by a research assistant to arrange a time and place for the interview. Participants will be interviewed after their 9-month participation in the trial has ended.

### Data monitoring

Data collection will be monitored by an external monitoring agency experienced in NHS Research and Development (R&D) processes. The following will be monitored: collection of the JRS and BSDI© at each time point, identification of adverse events not recorded and the appropriate recording of telephone calls from patients requesting appointments. The first monitoring visit will take place shortly after recruitment commencing to identify issues early into the study and resolving these promptly. A single further follow-up visit will be scheduled to take place 6 months after the first.

### Patient safety

Patient safety will be monitored by the specialist research nurse treating patients and the research fellow. Participants will be automatically booked back into the nurse-led botulinum toxin clinic at the end of the trial period by the trial co-ordinator. Reasons for not initiating treatment during participation in the trial will be recorded for any patients who have not attended for the full duration and all patients will continue to receive standard care.

### Sample size

Sample size for multilevel modelling (MLM) was estimated by simulating a range of scenarios using the software Power and Precision™ (Version 4.1; [32]). Using a large ICC of 0.3 [33], time as nested within participants, and setting power at 80 %, it was estimated that between 92 and 230 participants would be required with small to medium effects (Table 2). Based on a 79 % completion rate [9] this would mean that between 112 (56 per group) and 278 (139 per group) will need to be consented into the trial.

**Table 2:** Sample size estimation based on four scenarios with small to medium effect sizes

Scenario	Effect size (*d*)	ICC	Standard care	Intervention	Time points	Alpha	Tails	Power
Scenario 1	0.25	0.3	115	115	4	0.05	2	0.80
Scenario 2	0.3	0.3	80	80	4	0.05	2	0.80
Scenario 3	0.35	0.3	59	59	4	0.05	2	0.80
Scenario 4	0.4	0.3	46	46	4	0.05	2	0.81

The qualitative study assessing acceptability of the new service aims to recruit an initial sample of 10 participants with a stopping criterion of a further three interviews to confirm that data saturation has been achieved [34]. Data saturation is defined as the emergence of no new themes in relation to the research question.

### Statistical methods

All quantitative analyses will be undertaken in IBM SPSS Statistics Version 22.0 by the research fellow and trial statistician.

#### Changes over time

In order to explore stability in the primary outcome measures over the treatment period, and change in satisfaction, quality of life, mood and illness and treatment beliefs, multilevel models (MLM) will be employed. Time will be nested within participants and to check the assumption that scores within a participant are highly correlated, the first MLM will include no predictors and a scaled identity covariance type for both level 1 and level 2 in order to calculate the intraclass correlation coefficient (ICC). Models will be fitted with a first order autoregressive (AR1) covariance structure. Restricted Estimate Maximum Likelihood (REML) methods will be used as these are preferred for smaller samples [35].

Trial arm (0 = control, 1 = intervention), time (0, 1, 2) and the interaction between trial arm and time will be entered as fixed effects in each model, with participant identification number as a random effect. A significant interaction term will be interpreted as evidence for differential treatment effectiveness. Pairwise comparisons will be performed in order to establish where the significant differences lie, using estimated marginal means and standard errors. Standardised adjusted effect sizes for group differences at each time point will be calculated using Hedges’ *g* along with 99 % confidence intervals (as *p* < 0.01) using the formula provided by Turner and Bernard [36]. Hedges’ *g* includes a correction factor for small samples which, if absent, may lead to a less accurate and upwardly biased effect size. These effect sizes are interpreted in the same way as Cohen’s *d* [37] (small = 0.20, medium = 0.50, large = 0.80).

#### Difference in mean costs

Costs of services used by each participant will be estimated from the quantities of each type of resource used, multiplied by the unit cost. A total cost per case per patient will be calculated based on all of the above which in turn will allow for an average cost per case per trial arm for treatment and the control group, presented with rates of significance of difference between arms.

A cost utility analysis will be performed combining the cost data with the primary clinical outcome measures for functional disability (BSDI©) and disease severity (JRS). A further cost utility analysis will be performed on quality of life (CDQ-24), a secondary clinical outcome measure for the trial.

Cost per unit of therapeutic change, whether this be clinical improvement, reduced disability, change in quality of life or increased patient satisfaction will be calculated.

As part of the economic evaluation, non-parametric bootstrapping will be used to develop confidence intervals around the incremental cost-effectiveness ratio based on costs and associated effectiveness data (functional disability, disease severity, quality of life and patient satisfaction). This process also generates acceptability curves to illustrate the uncertainty associated with the estimate of costs and effects combined and (probabilistic) estimates of affordability given potentially different decision-maker cost thresholds.

#### Acceptability of the service

The questionnaire used to capture participants’ views of acceptability of the intervention will be validated after data has been collected. Recognised and widely employed questionnaire validation methods will be used to assess for internal consistency, concurrent, discriminant and predictive validity.

### Qualitative analysis

The semi-structured interviews will be digitally recorded with the participant’s consent and transcribed verbatim by a professional transcribing company. Any information which could identify a participant will be anonymised. The data generated from these semi-structured interviews will be analysed using framework analysis [38]; a method for identifying, analysing and reporting patterns within data, using NVivo Software (Version 10) [39]. This method allows themes to be identified across a dataset and is appropriate for use in studies with a focused research question, as in the case of this study. Framework analysis involves searching across a data set to find the key issues and themes following five steps: (1) familiarising yourself with your data, (2) identifying a thematic framework, (3) indexing, (4) charting, and (5) mapping and interpretation [38].

In addition to this six-phase process, four validity criteria will be employed: (1) an audit trial which involves detailed quotes from the participant’s transcripts to provide evidence for the interpretation offered, (2) a peer panel: an auditor will be asked to go through randomly selected sections of transcripts to confirm the pattern of analysis, (3) the researchers will attempt to recognise their own values, interests and views and the role that they may play in their understanding of the transcripts. Doing this can help the reader to interpret the researcher’s data and analysis. A reflective diary will be kept by the researcher, where notes are kept about initial thoughts and feelings, the main points that arise in the interview and any factors that the researcher felt influenced the interviewee. These will be taken into account throughout the analysis process, and (4) an independent audit: an independent auditor familiar with framework analysis will be asked to check the validity of the ‘final report’.

## Research ethics approval

The trial has received full NHS Research Ethics Committee (REC) approval (REC reference: 15/LO/0439) from London – Queen Square REC. Patients must provide written informed consent to take part in the study. Any data collected from the study participants will be anonymised and no identifying information will be used in publications.

## Dissemination

The study results will be disseminated to relevant healthcare professionals via appropriate pre-identified healthcare journals. The results will also be disseminated to the public through the publicly available clinical trials database clinicaltrials.gov.uk. The results will also be presented at relevant scientific conferences.

## Patient and public involvement

Two patients with blepharospasm and hemifacial spasm have been invited to join the study management group. These patient members have provided valuable feedback on study materials (e.g. information sheets, consent forms) and acceptability of study measures and will help with interpretation and dissemination of the research findings. The trial will also be presented at a Blepharospasm Patient Study Day organised by MEH in November 2015 to gain feedback from patients about the acceptability of the intervention from their perspective.

## Discussion

Given the shift away from the paternalistic model of healthcare, it is important to provide patients with the tools to take a more active role in knowing and managing their health. This trial will enable patients with dystonia to control the timing of their appointments and ultimately manage their own symptoms. This study is the first RCT to assess the effectiveness of patient-initiated treatment model for patients with blepharospasm and hemifacial spasm, whilst improving patient satisfaction with care, in comparison to treatment as usual.

The evaluation of this new service will use a rigorous mixed-methods design to explore whether empowering patients to initiate their own appointments allows for a more stable pattern of disease severity and symptoms, whilst improving patient satisfaction and acceptability with their care, and quality of life. The costs of delivering the service, and the impact it has on other healthcare use will be measured and compared in relation to control participants. The authors do not anticipate any safety concerns for the trial; however, appropriate risk assessment and monitoring procedures have been implemented.

If the new model is shown to be effective in maintaining a more stable pattern of disease severity, symptoms and disability, and at the same time being satisfactory and acceptable to patients, whilst being cost-effective, this will have significant implications for service organisation across the NHS.

## Trial status

Patient recruitment for this trial began in August 2015.

## Abbreviations

A&E: accident and emergency
AE: adverse event
AR1: first order autoregressive structure
BSDI: Blepharospasm Disability Index
CDQ: Craniocervical Dystonia Questionnaire
CSRI: Client Service Receipt Inventory
CSQ: Client Satisfaction Questionnaire
EPR: electronic patient records
HADS: Hospital Anxiety Depression Scale
ICC: intraclass correlation coefficient
ICMJE: The International Committee of Medical Journal Editors
IPQ-R: Illness Perceptions Questionnaire Revised
JRS: Jankovic Rating Scale
MEH: Moorfields Eye Hospital
MLM: multilevel modelling
NHS: National Health Service
RCT: randomised controlled trial
REC: Research Ethics Committee
REML: Restricted Estimate Maximum Likelihood
R&D: Research and Development department
SAE: serious adverse event
UK: United Kingdom
VAS: visual analogue scale
