# Erratum to: ‘Effectiveness and cost-effectiveness of a patient-initiated botulinum toxin treatment model for blepharospasm and hemifacial spasm compared to standard care: study protocol for a randomised controlled trial’

**DOI:** 10.1186/s13063-016-1502-2

**Published:** 2016-07-20

**Authors:** Sadie Wickwar, Hayley McBain, Stanton P. Newman, Shashivadan P. Hirani, Catherine Hurt, Nicola Dunlop, Chris Flood, Daniel G. Ezra

**Affiliations:** Centre for Health Services Research, School of Health Sciences, City University London, London, UK; Moorfields Eye Hospital, London, UK; Community Health Newham, East London Foundation Trust, London, UK

## Erratum

Unfortunately, the original version of this article [1] contained an error. In the Trial registration section the Clinicaltrials.ogv ID was included incorrectly as NCT102577224. The Correct ID is NCT02577224. There will also be an update to correct this error.
